# Hypoxia induced hERG trafficking defect linked to cell cycle arrest in SH-SY5Y cells

**DOI:** 10.1371/journal.pone.0215905

**Published:** 2019-04-24

**Authors:** Damodara Reddy Vaddi, Lin Piao, Shakil A. Khan, Ning Wang, Nanduri R. Prabhakar, Jayasri Nanduri

**Affiliations:** Institute for Integrative Physiology, Center for Systems Biology of O_2_ Sensing, Biological Sciences Division, The University of Chicago, Chicago, Illinois, United States of America; Duke University School of Medicine, UNITED STATES

## Abstract

The alpha subunit of the voltage gated human ether-a-go-go-related (hERG) potassium channel regulates cell excitability in a broad range of cell lines. HERG channels are also expressed in a variety of cancer cells and control cell proliferation and apoptosis. Hypoxia, a common feature of tumors, alters gating properties of hERG currents in SH-SY5Y neuroblastoma cells. In the present study, we examined the molecular mechanisms and physiological significance underlying hypoxia-altered hERG currents in SH-SY5Y neuroblastoma cells. Hypoxia reduced the surface expression of 150kDa form and increased 125kDa form of hERG protein expression in the endoplasmic reticulum (ER). The changes in protein expression were associated with ~50% decrease in hERG potassium conductance. ER retention of hERG 125kDa form by CH was due to defective trafficking and was rescued by exposing cells to hypoxia at low temperatures or treatment with E-4031, a hERG channel blocker. Prolonged association of hERG with molecular chaperone Hsp90 resulting in complex oligomeric insoluble aggregates contributed to ER accumulation and trafficking defect. Hypoxia increased reactive oxygen species (ROS) levels and manganese (111) tetrakis (1methyl-4-pyridyl) porphyrin pentachloride, a membrane-permeable antioxidant prevented hypoxia-induced degradation of 150kDa and accumulation of 125kDa forms. Impaired trafficking of hERG by hypoxia was associated with reduced cell proliferation and this effect was prevented by antioxidant treatment. These results demonstrate that hypoxia through increased oxidative stress impairs hERG trafficking, leading to decreased K+ currents resulting in cell cycle arrest in SH-SY5Y cells.

## Introduction

The human ether-a-go-go-related gene (hERG), the α subunit of a voltage gated potassium channel encodes a rapidly activating delayed rectifier current (I_kr_) [1]. Congenital or drug induced disruptions of the hERG channel cause long QT syndrome type 2 (LQT2), a cardiac disorder that predisposes affected individuals to ventricular arrhythmias and cardiac arrest [2, 3]. A majority (~80%) of the hERG missense mutations thus far studied are due to defective trafficking of hERG protein to the cell surface [4–7]. hERG protein synthesized in the endoplasmic reticulum (ER), as an immature core glycosylated protein (cg) of about 125kDa, is exported to the Golgi apparatus for complex glycosylation and eventually inserted into the plasma membrane as fully glycosylated mature protein (fg) of ~150kDa [8, 9]. HERG maturation and trafficking of the protein to the cell surface is regulated by the molecular chaperone Hsp90, which protects proteins from misfolding and degradation [10].

HERG potassium channels, originally identified as promoters of cardiac action potential repolarization, are now shown to serve as regulators of proliferation and apoptosis in cancer cells [11–13]. The hERG gene and protein are overexpressed in various cancer cell lines including epithelial, neuronal, leukemic and connective tissue and are absent in the corresponding non-cancerous cells [14]. Silencing hERG or selective hERG channel blockade by pharmacological inhibitors lead to reduced proliferation, cell cycle arrest and increased apoptosis in cancerous cells [15, 16] [17].

Hypoxia, a hallmark of tumors, influence both tumor progression and resistance to therapy [18]. Continuous hypoxia (CH) lasting several days alters gating properties of hERG currents in neuroblastoma cells [19]. We previously reported that CH results in decreased protein expression and hERG current density in HEK cells that stably express hERG protein [20]. Although hERG channel activity has been studied in neuroblastoma cells [19], the molecular mechanisms and the physiological significance of CH-evoked changes in hERG currents is not known. Consequently, in the present study, we examined the effects of CH on hERG protein expression and currents in SH-SY5Y neuroblastoma cells which express high abundance of endogenous hERG protein. Our results demonstrate that exposure of SH-SY5Y cells to 4days of CH decreased hERG surface protein expression and reduced hERG-dependent K+ conductance and these effects were due to defective trafficking. CH generated reactive oxygen species (ROS) contributes to misfolding of hERG protein in the ER resulting in prolonged association with molecular chaperone Hsp90, leading to defective trafficking. Moreover, CH-evoked hERG trafficking defect inhibited cell growth and proliferation of SH-SY5Y cells which was prevented by antioxidant treatment.

## Materials and methods

### Exposure of cell cultures to CH

Neuroblastoma (SH-SY5Y) cells were purchased from American Type Culture Collection (ATCC) (#CRL-2266). SH-SY5Y and HEK293 cells stably expressing the α subunit of the hERG channel were cultured in DMEM medium supplemented with 10% FBS, 100 U/ml penicillin and 100 mg/ml streptomycin and maintained at 37°C in 10% CO_2_. The cells were exposed to hypoxia (1.5% O_2_) for 2 and 4 days in a hypoxic chamber. In experiments involving drug treatments, cells were pre-incubated for 30 min with either drug or vehicle before hypoxia exposure.

### Measurement of hERG K^+^ currents

hERG currents were monitored at room temperature by whole-cell configuration of the patch clamp technique as described previously [10]. hERG currents were recorded using patch pipettes filled with (in mM): K^+^-aspartate 100, KCl 20, MgCl_2_ 2, CaCl_2_ 1, EGTA 10 and HEPES 10 (pH 7.2). The extracellular solution was (in mM): NaCl 140, KCl 5, MgCl 1, CaCl_2_ 1.8, HEPES 10 and glucose 10 (pH 7.4). Cells were depolarized from a holding potential of -80mV to +10mV in 10mV step range in a 2sec step current. Tail currents were recorded on repolarization to -90mV for 1sec. Maximal tail current amplitudes were normalized to cell capacitance to compute hERG tail current densities.

### Immunocytochemistry

Cells coated on cover slips were fixed with 4% formalin, blocked and permeabilized in 5% BSA and 0.1% triton X-100 for 30 minutes at RT. Permeabilized cells were incubated with anti-hERG (1/200 dilution, Alamone #APC-062) or anti cadherin (1/100 dilution, Abcam # AB6528) or anti-grp94 (1/50 dilution, Stressgen #SPA-850) for 2hrs at 37°C, washed with PBS and incubated with Alexa conjugated secondary antibody for 1hr at 37°C. Cells were mounted in DAPI containing media and analyzed using a Nikon fluorescence microscope.

### Immunoblot assay

Cells were solubilized at 4°C in lysis buffer (50mM Tris [pH 7.5], 150mM NaCl, 1mM EDTA, 1% Triton X-100) containing a protease inhibitor cocktail for 30 min at 4°C followed by sonication of 10 short bursts of 2sec and supernatant recovered by centrifugation at 13,000 g for 30 min. Protein concentrations were determined, and equal amounts of proteins were separated on a 6% SDS-PAGE gel and transferred to polyvinylidene difluoride membrane (PVDF). Membranes were blocked in 5% milk and immunoblotted with anti-hERG (1/1000 dilution, Alamone, Cat #APC-062) and anti-Hsp90 (1/1000 dilution, Stressgen # SPA835) antibody which were detected using HRP conjugated secondary antibodies followed by enhanced chemiluminescence (ECL) detection system (Bio-Rad). Tubulin (1/10,000 dilution, Sigma #T6199) was used as loading control Image densities were quantified using ImageJ (NIH) by integrating pixel densities of individual protein bands and normalized to tubulin

### Trypsin digestion and detergent extraction:

Membrane proteins from SH-SY5Y cells exposed to normoxia or CH were prepared using membrane extraction kit (Abcam, #a b6540) and resuspended in Tris buffer saline (50mM Tris-Hcl, pH 7.4 and 150mM NaCl). 10ug of protein was treated for 5min at room temperature with various concentrations of trypsin. The reaction was stopped by addition of trypsin inhibitor (ATi) to a final concentration of 1mg/ml and samples analyzed by immunoblots with anti-hERG antibody. For detergent extraction, cells were harvested in lysis buffer (50mM Tris-Hcl, pH 7.4 and 150mM NaCl, 1mM EDTA) with 0.5% triton X-100 and incubated at 4°C for 30min. Detergent soluble and insoluble proteins were separated by centrifugation at 100,00g for 20 min and analyzed by immunoblots.

### Measurements of ROS by fluorescence and aconitase activity

Cells plated onto poly-lysine coated cover slips were exposed to 4days of CH. CM-H_2_DCFDA 5-(and-6) chloromethyl 2’, 7’dichlorodihydrofluorescein diacetate; 25uM) was added to the cells 30 min prior to terminating the hypoxic exposure. Subsequently, cells were washed, mounted on glass slides and fluorescence images were taken. Fluorescence intensity >5 times background was defined as positive staining and the number of cells with positive staining was pooled across five fields in a given specimen. Aconitase activity was measured using an aconitase assay kit (Cayman Chemical Company; # 705502) as described. Protein concentration was estimated in the cell extracts using Bio-Rad protein assay kit. The enzyme activities were expressed as nmol/mg of protein.

### Cell growth and cell cycle analysis

For growth curve studies, cells were seeded in a 6 well microplate at a concentration of 1x10^3^ per well and cultured at normoxia or CH for 2 and 4 days. Cells were harvested and stained with trypan blue and live cells were counted using a hemocytometer. BrdU incorporation was performed in cells cultured at normoxia or CH for 48 hours following the standard protocol (Becton Dickinson) after a 30 min pulse with10 μmol/L BrdU. Cells were stained with Alexa 488 anti-BrdU (Invitrogen). For cell cycle analysis, cells exposed to normoxia and CH were collected by trypsinization and washed in phosphate-saline buffer. Cells were fixed in ice-cold ethanol overnight at 4°C and resuspended in propidium iodide (PI) stain buffer for 15–30 min at room temperature and subjected to flow cytometry analysis.

### Statistical analysis

Data are expressed as mean ± SEM from 3–5 independent experiments. Statistical analysis was performed by analysis of variance (ANOVA). The Wilcoxon-Mann-Whitney test was used for analysis of normalized data. *p* values < 0.05 were considered significant.

## Results

### CH downregulates hERG protein and hERG currents

**Fig 1:**
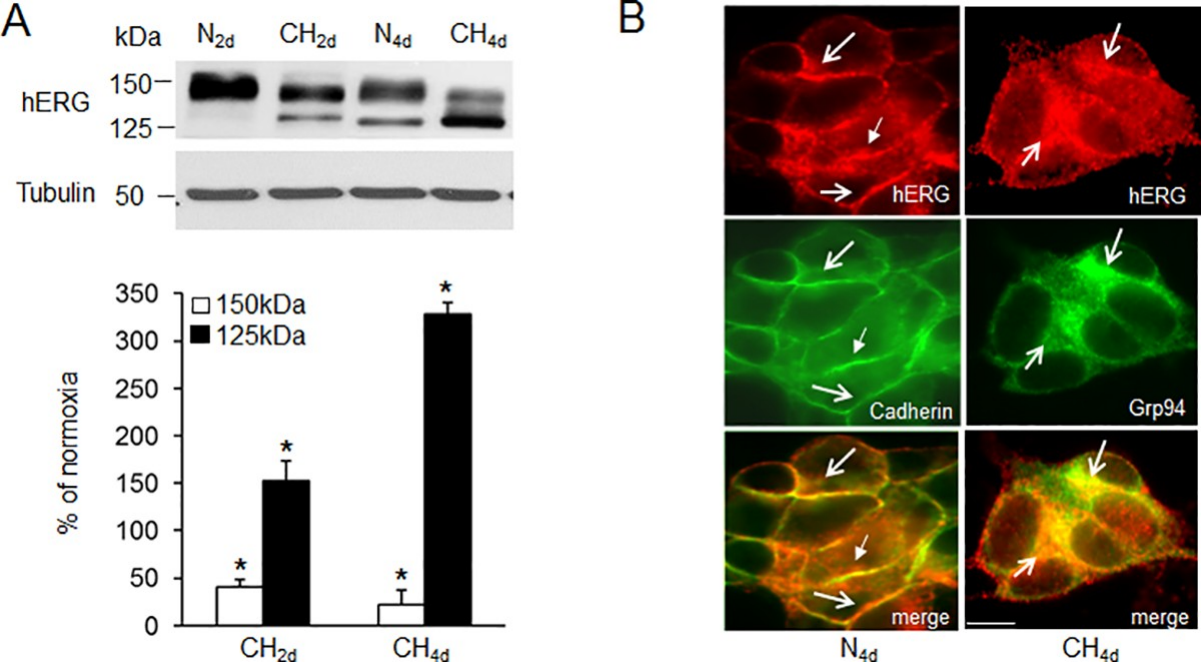
Effect of continuous hypoxia (CH) on hERG protein expression. A). Representative immunoblot of hERG protein in SH-SY5Y cells exposed to either normoxia (N) or 2 or 4 days of CH (1.5% O_2_) *(Top panel)*. Densitometric analysis (mean ± SEM) of hERG protein normalized to tubulin and expressed as percentage of normoxia (*Bottom panel*). n = 5 individual experiments. * denotes p <0.01 B) Immunolocalisation of hERG (red) with cadherin (green) and Grp94 (green) in cells exposed to normoxia (N) and CH_4d_ respectively. Arrows denote membrane localization and ER accumulation of hERG in N and CH cells respectively.

Immunoblot analysis showed that hERG protein consists of two forms, a 150kDa band and a 125kDa band in SH-SY5Y cells exposed to normoxia and CH respectively (Fig 1A). The specificity of the two bands was further confirmed by preadsorbing the diluted antibody with an excess of the peptide used to raise the antibody. Preadsorbtion completely inhibited the binding of antibody to 150kDa and 125kDa protein bands (S1A Fig). In addition, the antibody recognized the same two bands 150 and 125kDa only in HERG-transfected HEK 293 cells and not in untransfected HEK 293 cells (S1B Fig) as shown previously (4–6, 10, 20). Expression of the 150kDa hERG protein decreased by 40% with 2 days and by 80% with 4 days of CH (1.5% O_2_) as compared to normoxia (Fig 1A). In contrast, 2 and 4 days of CH increased the steady state levels of the 125kDa form by 170% and 325% respectively (Fig 1A). Immunocytochemical analysis showed that hERG protein is primarily localized to plasma membrane under normoxia as evidenced by co-localization with cadherin, a plasma membrane marker protein (Fig 1B). Four days of CH reduced the membrane expression with concomitant increase in hERG protein in the endoplasmic reticulum (ER) as indicated by dual labeling of cells with Grp94 an ER marker protein (Fig 1B). Based on these results, all further experiments were performed with 4 days of CH exposure, which produced maximal changes in hERG protein expression.

**Fig 2:**
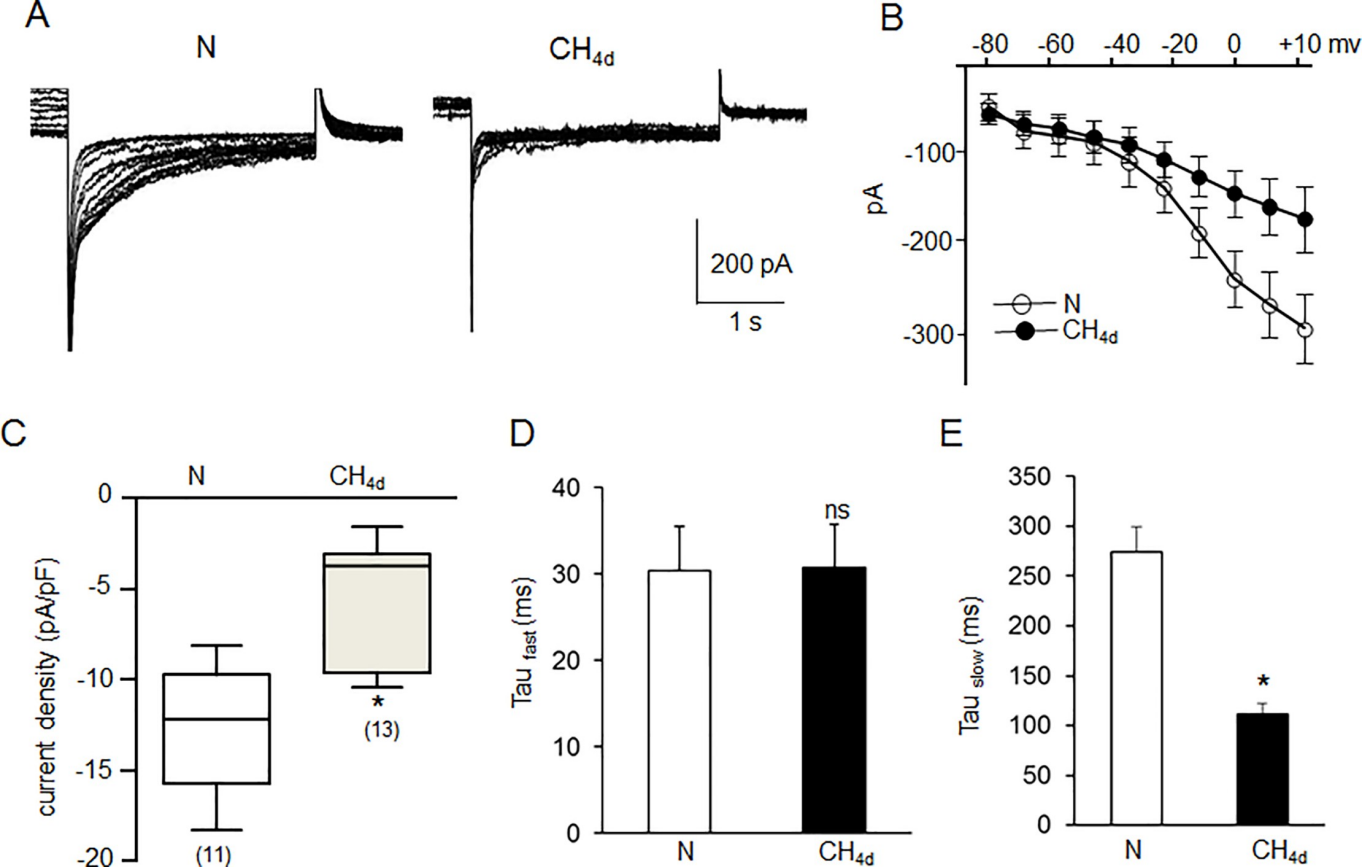
Effect of continuous hypoxia (CH) on hERG-mediated K+ conductance. A) Representative whole-cell currents recorded from cells under normoxia and in cells exposed to CH_4d_. B) Representative voltage-dependent properties of hERG currents in a control cell (N) and in a cell exposed to CH_4d_. C) Average data (mean ± SEM) of hERG current densities from cells exposed to normoxia (n = 11) and CH_4d_ (n = 13). Current density was derived by normalizing current amplitudes to cell capacitance. To give a measure of data dispersion, data are presented in box charts, whiskers marking the 5^th^ and 95^th^ percentile and the box determining the 25th and 75th percentile. Arithmetic means are represented by open squares. * denotes p <0.01. D & E) Fast and slow deactivation time constants were compared between normoxia and CH_4d_ exposed cells.

To assess whether changes in hERG protein by CH are reflected in hERG function, K^+^ currents were recorded in SH-SY5Y cells in the presence and absence of 1μM E-4031, a selective blocker of hERG K^+^ conductance [21]. The E-4031 blocked 70 ± 4% of the K^+^ current in normoxic cells (n = 15 cells; P<0.01), suggesting hERG channel mediates the majority of the observed K^+^ conductance. Fig 2A shows representative voltage-dependent properties of hERG currents in a control cell (N) and in a cell exposed to 4 days of CH (CH_4d_). Maximal tail current amplitude, which represents the relative proportion of channels that had been activated during depolarization was significantly lower in CH cells compared to controls (Fig 2B). Normalizing hERG current values to cell capacitances showed that hERG current density (pA/pF) decreased significantly with 4 days of CH (-4.63±11.14, n = 13) compared to controls (-12.54±1.22, n = 11) (Fig 2C). The fast and slow deactivation time constants were compared between normoxia and CH exposed cells (Fig 2D and 2E). The fast deactivation time constants were not different between the control group (31.7±5.8ms) and the CH group (32.5±5.0ms), whereas, slow deactivation time constants were significantly shortened in CH group (106.2±14.8ms) compared to the controls (268±29.6ms), suggesting CH not only reduced hERG current amplitudes but also altered the deactivation kinetics of hERG currents.

### CH impairs hERG trafficking

**Fig 3:**
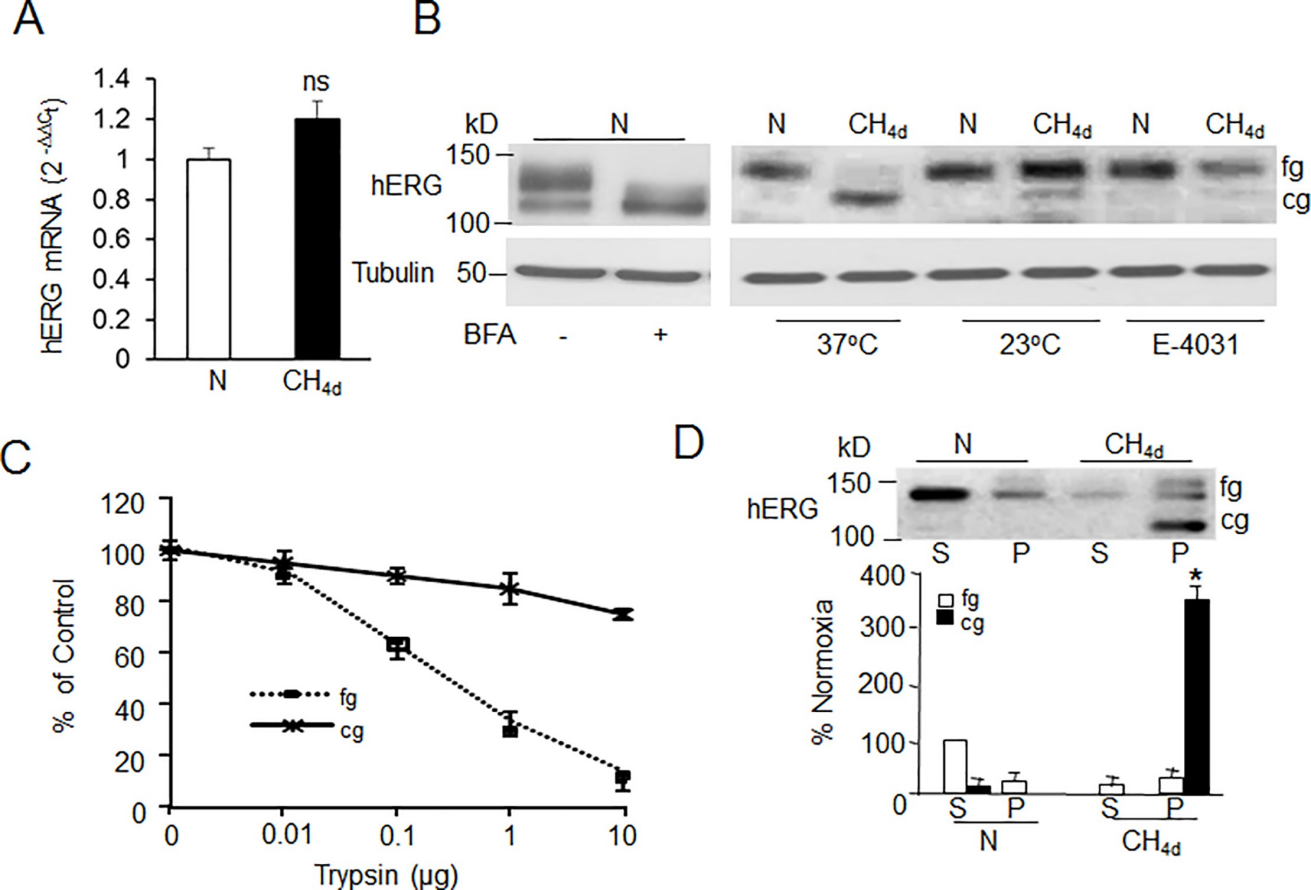
Continuous hypoxia (CH) impairs hERG trafficking. A) Quantitative real-time PCR analysis of hERG mRNA levels in SH-SY5Y cells exposed to normoxia (N) or CH_4d_. The data are mean ± SEM; n = 3 individual experiments. B) Representative immunoblot analysis of hERG protein in cells exposed to normoxia (N) with and without 5μM brefaldin treatment. Effect of temperature and antiarrhythmic drug (E-4031; 5μM) on hERG expression in normoxia and CH exposed cells. C) Trypsin sensitivity of 150kDa and 125kDa forms of hERG. Total cell membrane lysates from normoxia and CH exposed cells were treated with different concentrations of trypsin followed by immunoblotting with hERG antibody. Densitometric analysis (mean ± SEM) of hERG protein normalized to tubulin and expressed as percentage of control (without trypsin treatment). D) Detergent extractability of hERG from cell lysates exposed to normoxia and CH. Equal amounts of detergent soluble (S) and insoluble (P) proteins from normoxia and CH exposed cells were analyzed by immunblotting. Data are mean ± SEM. from 3 individual experiments. * denotes p <0.01.

ER accumulation of hERG could either be due to increased synthesis or due to trafficking defect. Analysis of hERG mRNA expression by real time RT-PCR showed that CH did not alter mRNA levels as compared to controls (Fig 3A) suggesting that defective trafficking rather than synthesis is involved. CH induced ER accumulation is reminiscent of effects induced by brefaldin A (BFA), a fungal metabolite that inhibits ER to Golgi transport. As shown by immunoblot and localization studies (Fig 3B and S2 Fig), treatment of SH-SY5Y cells under normoxic conditions with 5μm BFA, resulted in decreased surface expression of the 150kDa form with increased ER accumulation of the 125kDa form, mimicking the effects of CH. Exposure of cells to CH at low temperature (23°C) and treatment of cells with E-4031 (hERG channel blocker), known to rescue trafficking defects, completely restored the expression of the 150kDa form to normoxic levels further confirming that CH impaired hERG trafficking (Fig 3B).

### Misfolding of ER form contributes to CH impaired hERG trafficking

To determine if the CH induced trafficking defect is due to misfolding, we studied hERG channel folding during normoxia and CH by trypsin digestion experiment. [22]. Membrane fractions prepared from CH or normoxia exposed cells were treated with increasing concentrations (0.01 to 10ug/ml) of trypsin and hERG protein was analyzed by immunoblot assay. As shown in Fig 3C, under normoxic conditions, the 150kDa band was significantly digested with 1ug/ml of trypsin treatment. In contrast, the ER accumulated 125kDa form in CH exposed cells was quite resistant to 10ug/ml of trypsin. The trypsin resistance could be due to significant aggregation of the misfolded hERG channel protein in the ER by CH. Aggregated proteins become resistant to detergent extraction [23]. To determine if CH leads to accumulation of aggregated hERG, cell lysates were separated into detergent soluble (S) and detergent insoluble (P) fractions as described in methods. The 150kDa form in normoxic lysates was predominantly in the detergent-soluble fraction (S in Fig 3D). In contrast, the ER retained 125kDa form from CH cells partitioned to the detergent insoluble fraction of the cell lysate (P in Fig 3D).

### Involvement of ROS in CH-impaired hERG trafficking

**Fig 4:**
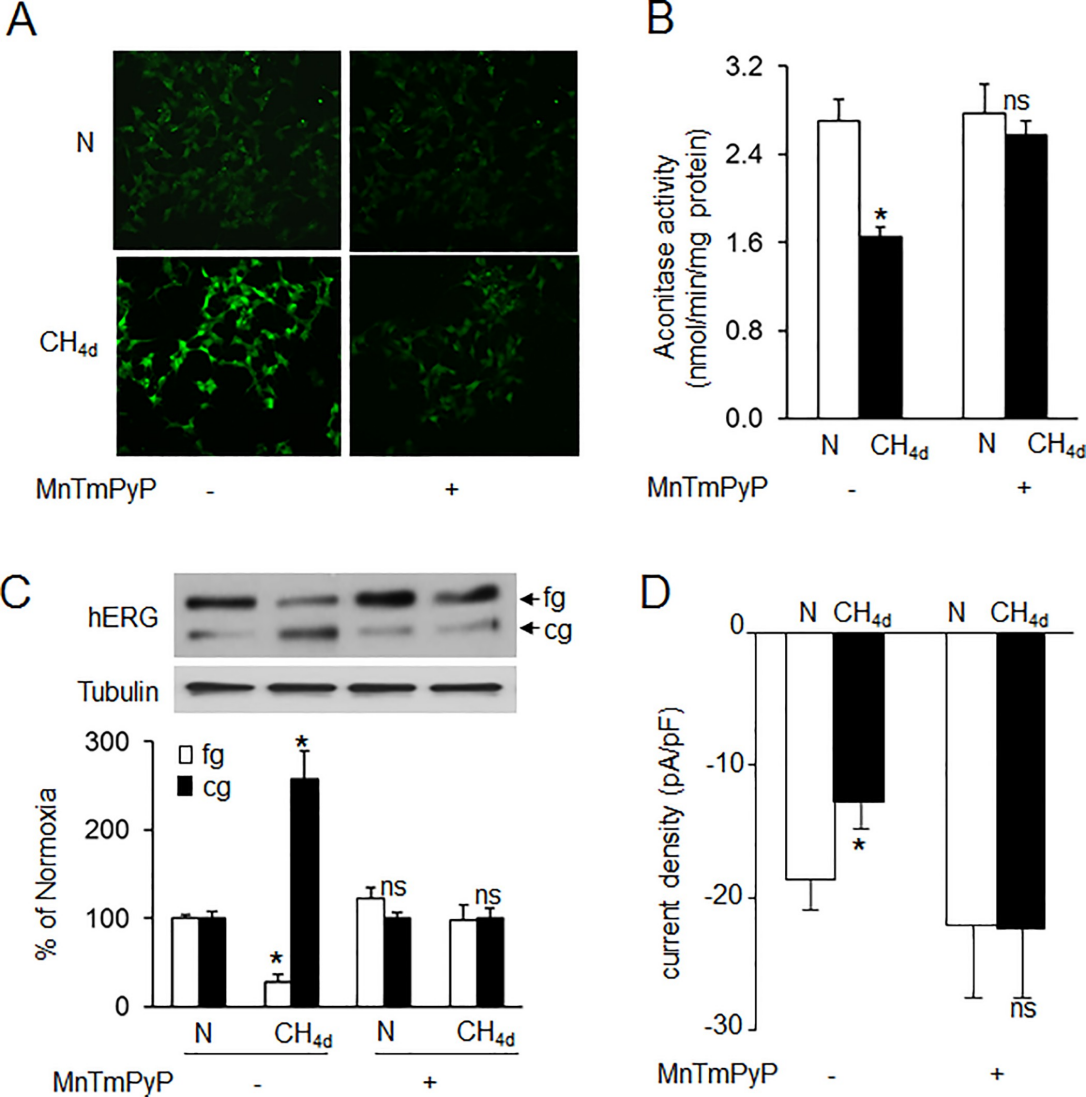
Role of reactive oxygen species (ROS) in continuous hypoxia (CH)-induced hERG trafficking defect. Analysis of ROS generation in SH-SY5Ycells exposed to normoxia (N) or CH_4d_ with and without MnTmPyP (50μM), a membrane permeable ROS scavenger as measured by **A)** CM-H_2_DCFDA flourescence and B) cytosolic and mitochondrial aconitase activity. Data shown are mean ± SEM from 3 independent experiments. C) Representative immunoblot (*top panel*) and densitometric analysis (*bottom panel*; mean ± SEM; n = 3 individual experiments) of hERG protein with and without MnTmPyP treatment and D) HERG current densities measured in cells exposed to normoxia (N) or CH_4d_ with and without MnTmPyP treatment. *denotes p <0.01. ns = not significant from control (N); p> 0.05.

We previously reported that reactive oxygen species (ROS) generated by CH decreases hERG current density and protein expression in HEK cells stably expressing hERG protein [20]. To examine the potential role of ROS in CH induced effects on hERG currents and protein in SH-SY5Y cells, we first determined cellular ROS levels in CH exposed cells by two approaches: one by staining of cells with H_2_DCFDA, a cell permeable indicator of ROS that is fluorescent upon oxidation and the other by determining the aconitase enzyme activity, an established biochemical marker of ROS in cytosol and mitochondrial fractions. ROS levels were significantly elevated in CH exposed cells as evidenced by an increase in the number of fluorescent positive cells (~70%) (Fig 4A) and decreased aconitase activity in the cytosolic and mitochondrial fractions (Fig 4B). Treatment of cells with MnTmPyP (10μM), a membrane permeable ROS scavenger prevented increased ROS levels and blocked CH-induced changes in hERG protein and restored hERG currents (Fig 4C and 4D).

### Modulation of hERG interaction with Hsp90 by ROS leads to hERG trafficking defect by CH

**Fig 5:**
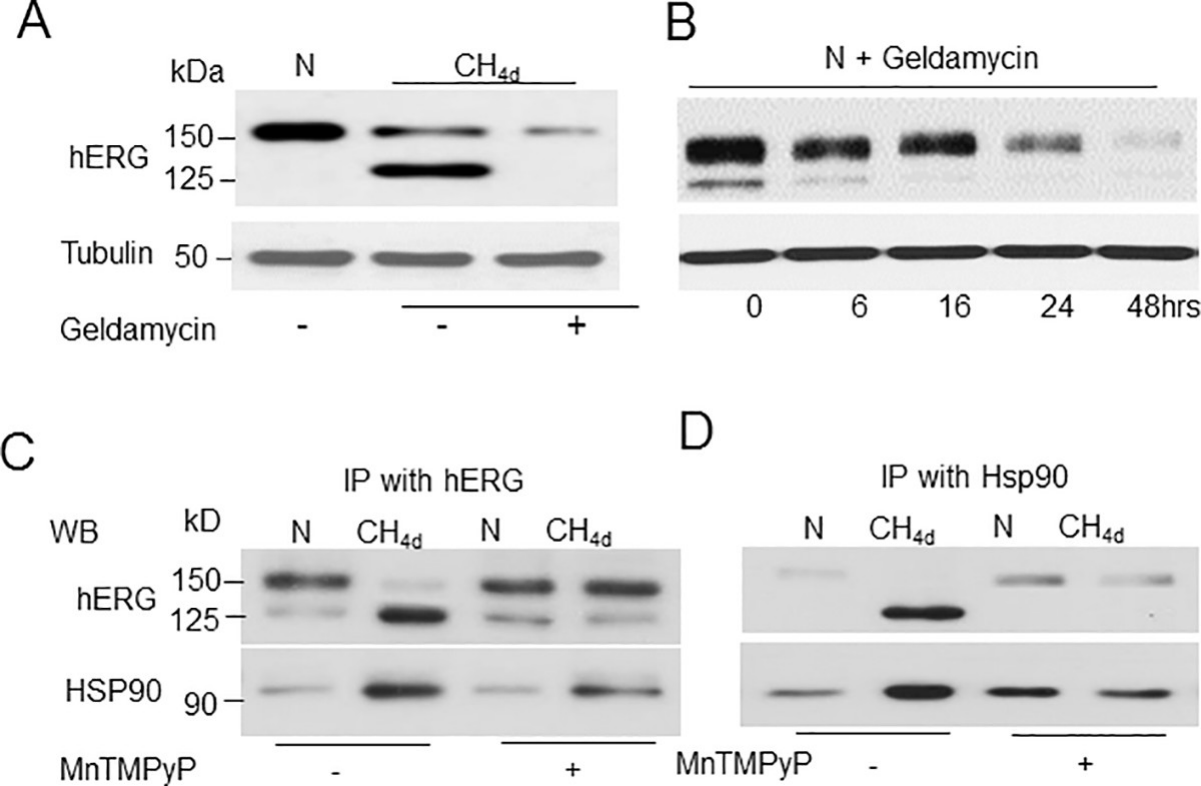
Effect of continuous hypoxia (CH) on hERG interaction with Hsp90. A) Representative immunoblot showing the effect of geldnamycin (1μg/ml) on hERG protein expression in cells exposed to normoxia (N) and CH_4d_. B) Time course of geldnamycin effect on hERG protein under normoxic conditions. C-D) Cross-linked hERG-Hsp90 complexes immunoprecipitated with anti-HERG (C) or anti-Hsp90 (D) antibodies and analyzed by immunoblotting in cells exposed to normoxia or CH_4d_ with and without MnTmPyP treatment. The 125kDa from of hERG co-immunoprecipitatied with Hsp90 in CH exposed cells which was blocked with MnTmPyP treatment. Data presented are mean ± SEM. from 3 individual experiments.

We then investigated how ROS contributes to CH-induced hERG trafficking defect. HERG maturation and trafficking through the ER is tightly regulated by its interactions with molecular chaperones Hsp90 and Hsp70 [10]. Trafficking defective hERG mutant proteins that are unable to fold correctly often exhibit prolonged rather than transient association with Hsp90, a property that may contribute to its retention in the ER. To determine if CH prolonged the association of hERG with Hsp90 chaperone, we treated cells with geldanamycin, a specific inhibitor of Hsp90 binding and analyzed hERG expression. Geldanamycin treatment under CH conditions blocked accumulation of the 125kDa form with significant decrease of 150kDa form (Fig 5A). Previous studies have shown that blocking Hsp90 binding to hERG by geldanamycin resulted in decreased surface expression of 150kDa form. In fact, as shown in Fig 5B, geldanamycin decreased 150kDa form in a time dependent manner. To further confirm that CH altered hERG interaction with Hsp90, we performed co-immunoprecipitation experiments. Since Hsp90 interacts transiently with proteins, hERG-hsp90 complexes were isolated by immunoprecipitation with anti-hERG or anti-Hsp90 antibodies after crosslinking with chemical cross linker DSP. HERG antibody immunoprecipitated 150kDa form under normoxic conditions whereas only 125kDa form was observed under CH. (Fig 5C). The 125kDa form was stably bound to Hsp90 in CH exposed cells which was blocked with MnTmPyP treatment (Fig 5C). Similar results were obtained when hERG-Hsp90 complexes were immunoprecipitated with Hsp90 antibody (Fig 5D).

### CH-induced hERG trafficking defect inhibits cell growth and proliferation

**Fig 6:**
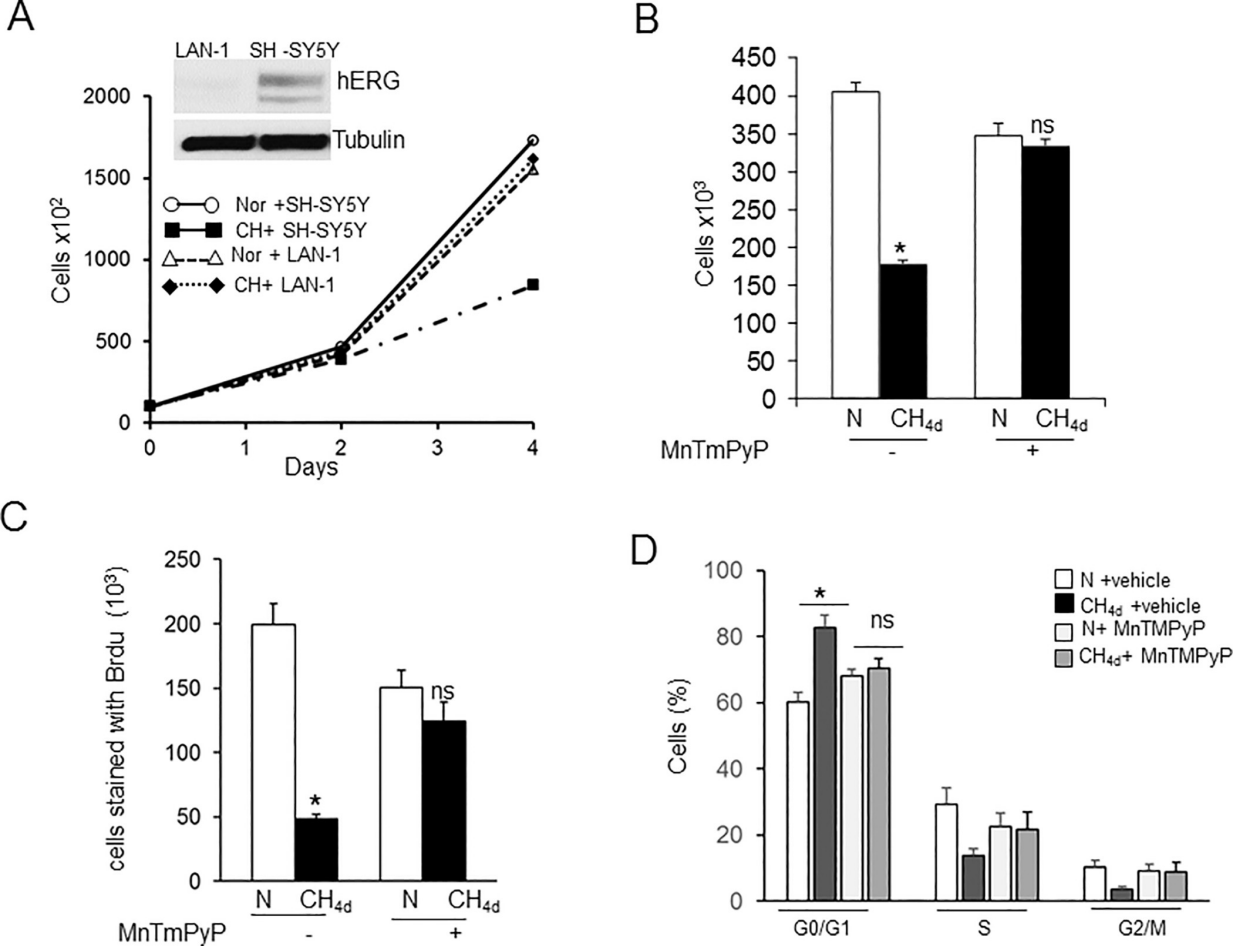
HERG downregulation by continuous hypoxia (CH) inhibited cell growth and proliferation. A) Growth of SH-SY5Y and LAN-1 cells exposed to normoxia (N) or CH_4d_ monitored as described in methods (inset shows hERG protein expression), B) Effect of MnTmPyP treatment on SH-SY5Y cell proliferation under normoxic (N) and hypoxic (CH_4d_) conditions, C) Changes in BrdU incorporation and D) Cell cycle analysis of cells exposed to normoxia and CH_4d_ by flow cytometry. Results are averaged from 3 independent experiments (mean ± SEM).

Hypoxia as well as selective blockade of hERG channels reduce cell proliferation of neuroblastoma cells [16, 18]. We examined the putative role of CH-induced hERG trafficking defect in cell proliferation. Growth curve of cells was determined in cells exposed to normoxia and CH. 4days of CH significantly impaired proliferation (by 50%) as compared to normoxia (Fig 6A). CH had no significant effect on the growth curve of neuroblastoma LAN-1 cells which do not express hERG [24]. Antioxidant treatment which blocked CH-induced hERG downregulation rescued SH-SY5Y cell proliferation to controls (Fig 6B).

To further confirm that CH inhibited cell proliferation, cells were stained with BrdU (thymidine analog, 5-bromo-2-deoxyuridine) a marker for cell proliferation. As shown in Fig 6C, percentage of cells stained with BrdU decreased significantly with CH (19 ± 2%) compared to normoxia (52±5%). MnTmPyP treatment showed a modest decrease in the number of BrdU stained cells under normoxic conditions and CH had no further effect, consistent with the results of growth curve analysis. Previous studies have shown that reduced cell growth by hERG downregulation is due to cell cycle arrest in the G0/G1phase [16, 24]. We therefore assessed cell cycle progression by analyzing the DNA content of cells with propidium iodide staining followed by flow cytometric method. Cell cycle analysis revealed significant accumulation of cells in the G0/G1phase following CH (82 ± 4%) compared to normoxia (60% ± 6%; Fig 6D). Cell numbers in the G0/G1 phase in MnTmPyP treated cells exposed to normoxia were 68%± 3% and CH had no further effect (70± 4%) (Fig 6D).

## Discussion

Major findings of the present study are: a) continuous hypoxia (CH) reduces membrane expression with concomitant increase in ER accumulation of hERG protein in SH-SY5Y cells, b) reduced membrane expression was due to defective trafficking of hERG protein from ER , c) the effects of CH on hERG protein were associated with reduced hERG K+ conductance and inhibition cell proliferation, and d) CH increased ROS abundance and anti-oxidant treatment prevented the trafficking defect and restored the membrane expression of the protein, K+ conductance and cell proliferation.

The following observations demonstrate that CH-induced hERG trafficking defect is due to ER retention of the misfolded 125kDa form stably bound to molecular chaperone Hsp90: a) CH-induced trafficking defect was rescued by culturing cells at low temperatures and E-4031 (pharmacological chaperone known to rescue trafficking defect) [5, 25, 26], b) hERG 125kDa form was resistant to trypsin digestion as opposed to 150kDa form which is sensitive to trypsin suggesting that CH results in misfolding of the hERG protein form in the ER, c) misfolding contributed to prolonged association of 125kDa band with molecular chaperone Hsp90 as evidenced by coimmunoprecipitation experiments and d) resistance of 125kDa form to detergent extraction suggests significant aggregation and ER retention similar to that observed with trafficking-deficient LQT2 mutants (hERG R752W and G601S). Defective trafficking of hERG is increasingly recognized as an important mechanism in hERG channel dysfunction in long QT syndrome (LQT2) [5–8] and our results suggest that the same mechanism seems to contribute to CH induced downregulation of surface membrane form of hERG in neuroblastoma cells.

Our studies provide further mechanistic insight into how CH alters hERG protein expression. Oxidative stress is a major component of several pathophysiological conditions. Tumor cells exhibit higher levels of ROS than normal cells and aberrant ROS production has been implicated in triggering several signaling pathways in cancer cells [27–30]. The following observations demonstrate that ROS signaling mediates hERG misfolding resulting in defective trafficking: a) ROS levels were elevated in CH exposed neuroblastoma cells as evidenced by increased DCFDA staining as well as decreased aconitase activity in the cytosolic and membrane fractions, b) MnTmPyP, a membrane permeable ROS scavenger prevented CH-induced ROS generation as well as hERG degradation by restoring proper folding of the ER form and its interaction with Hsp90 leading to cell surface expression. How might ROS contribute to hERG defective trafficking by CH? ROS could directly oxidize hERG’s numerous residues both in the PAS domain at the N-terminus or C-terminal cysteines that are potentially susceptible to redox modification [22]. This could result in a conformational change resulting in misfolding and altered hERG-chaperone interactions. Alternatively, ROS may regulate downstream links in signal transduction pathways associated with ER chaperones /calcium binding proteins required for subunit stability and assembly of hERG channels. However, further studies are needed to assess this possibility.

We and others [20, 31] reported in HEK293 cells stably expressing hERG protein, CH reduced 150kDa form but did not result in ER retention. The discrepancy between the current and the previous studies could be that endogenous hERG trafficking in SH-SY5Y cells may be regulated by other factors. Indeed such a possibility is supported by recent studies which identified a splice variant hERG1b with a truncated N-terminus and a unique sequence of 29 residues in SH-SY5Y cells [32]. Tumor cells preferentially express hERG1b which forms heterotetramers with hERG1a and is involved in the trafficking of hERG to the plasma membrane [33]. The precise role of hERG1b in CH induced hERG trafficking defect remains an open question.

The results of the present study support the hypothesis that hypoxia induced hERG trafficking defect inhibits cell growth of SH-SY5Y cells, which is due to cell cycle arrest. Although hypoxia-induced cell cycle arrest may seem paradoxical to its documented role in tumor growth, several studies suggest that tumor hypoxia is also associated with resistance to radiation therapy and chemotherapy by inhibiting cell proliferation in a variety of transformed cells. While contribution of oxygen–regulated hypoxia-inducible factors, HIF-1α [34] to cell cycle arrest has been observed in a wide spectrum of solid tumors in vitro, our study suggests hERG as another major molecular mediator of hypoxia-induced cell cycle arrest in neuroblastoma cells.

## Supporting information

S1 Fig Specificity of hERG antibody. Representative immunoblots showing the specificity of the two hERG protein bands (150 and kDa). A) Diluted hERG antibody preadsorbed with excess of the immunogen (provided with the antibody) overnight was used for immune blot of SH-SY5Y cells exposed to normoxia (N) or 2days of hypoxia (CH_2d_) and compared to unadsorbed antibody (left two lanes). B) HERG protein expression in HEK cells stably transfected with hERG plasmid subjected to normoxia (N) or 1day of hypoxia (CH_1d_) and compared with non-transfected HEK cells. Tubulin protein expression was used as a loading control in A and B.
(TIF)

S2 Fig Effect of brefaldin treatment. Immunolocalization of hERG (red) with cadherin (green) and Grp94 (green) in SH-SY5Y cells under normoxic conditions with and without brefeldin treatment. Arrows denote membrane localization and ER accumulation of hERG without and with brefeldin treatment respectively.
(TIF)
