# Correction: Hypoxia induced hERG trafficking defect linked to cell cycle arrest in SH-SY5Y cells

**DOI:** 10.1371/journal.pone.0297301

**Published:** 2024-01-11

**Authors:** Damodara Reddy Vaddi, Lin Piao, Shakil A. Khan, Ning Wang, Nanduri R. Prabhakar, Jayasri Nanduri

The N MnTmPyP- panel in Fig 4A of this article [1] was erroneously reused to represent the N MnTmPyP+ panel in this figure with alteration to contrast/brightness. The N MnTmPyP+ panel is corrected in the updated Fig 4 provided with this notice. Original image data underlying the results in Fig 4A are provided in S1 File.

The hERG panel in S1A Fig contains a discontinuity between lanes 2 and 3. The membrane was cut vertically between these lanes, the two halves were incubated in antibodies separately, and then placed together for imaging. S1 Fig and the legend are updated to delineate the two pieces of membrane. Original image data underlying the results in S1A Fig are provided in S2 File.

In addition, the article’s Data Availability statement is hereby updated to: The original underlying data to support all results in the article and Supporting Information files are available from the corresponding author, except for the original images underlying the hERG panel in Fig 3B and Hsp90 panel in Fig 5C which are no longer available.

**Fig 4:**
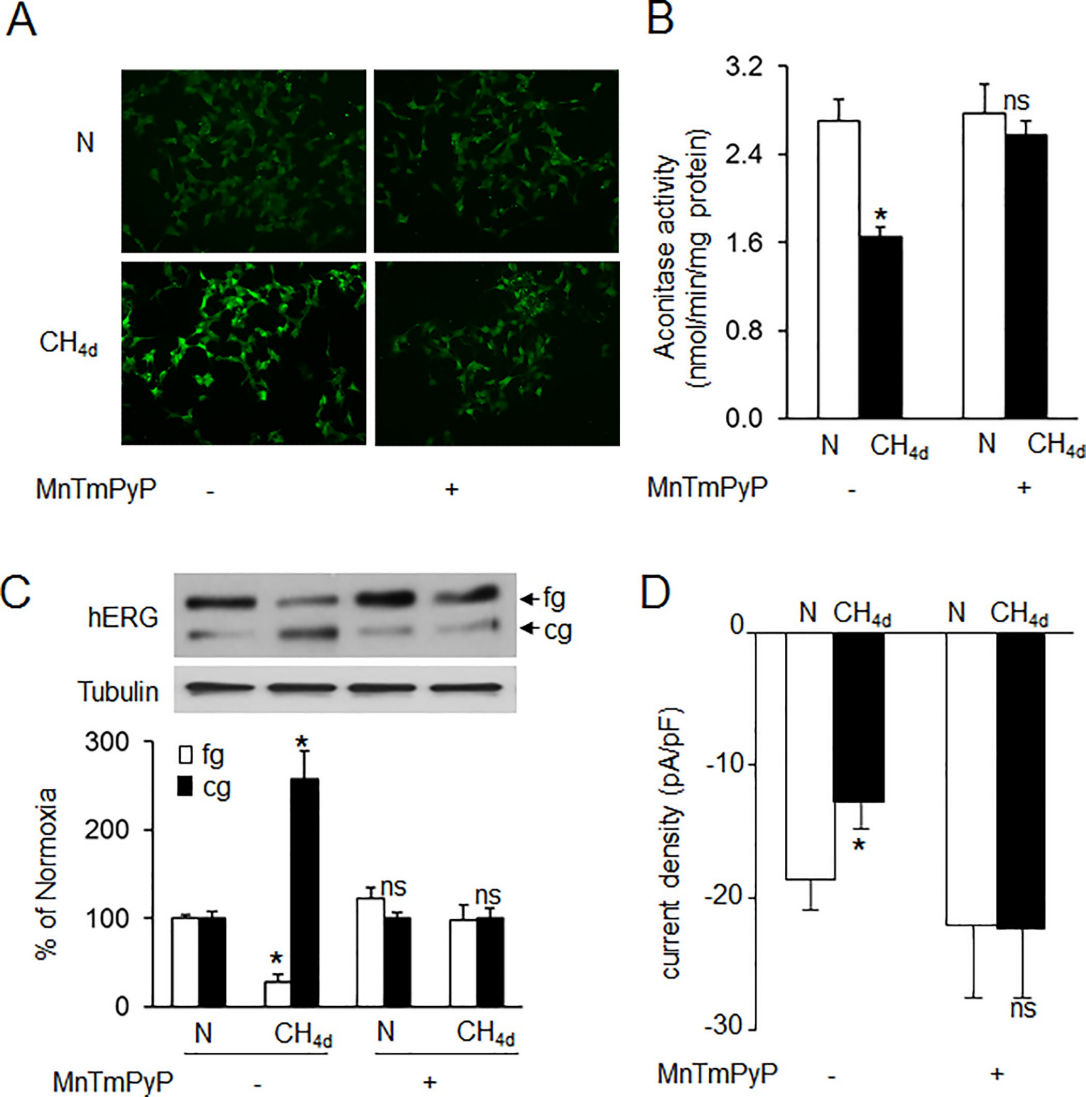
Role of reactive oxygen species (ROS) in continuous hypoxia (CH)-induced hERG trafficking defect. Analysis of ROS generation in SH-SY5Ycells exposed to normoxia (N) or CH_4d_ with and without MnTmPyP (50μM), a membrane permeable ROS scavenger as measured by **A)** CM-H_2_DCFDA fluorescence and B) cytosolic and mitochondrial aconitase activity. Data shown are mean ± SEM from 3 independent experiments. C) Representative immunoblot (*top panel*) and densitometric analysis (*bottom panel*; mean ± SEM; n = 3 individual experiments) of hERG protein with and without MnTmPyP treatment and D) HERG current densities measured in cells exposed to normoxia (N) or CH_4d_ with and without MnTmPyP treatment. *denotes p <0.01. ns = not significant from control (N); p> 0.05.

## Supporting information

S1 Fig Specificity of hERG antibody. A) Representative immunoblots showing the specificity of the two hERG protein bands (150 and kDa) in SH-SY5Y cells exposed to normoxia (N) or 2days of hypoxia (CH_2d_) probed with hERG antibody (left two lanes) or with hERG antibody preadsorbed overnight with excess of the immunogen (provided with the antibody) (right two lanes). B) HERG protein expression in HEK cells stably transfected with hERG plasmid subjected to normoxia (N) or 1day of hypoxia (CH_1d_) and compared with non-transfected HEK cells. Tubulin protein expression was used as a loading control in A and B.
(TIF)

S1 File Image data underlying Fig 4A. (ZIP)

S2 File Image data underlying S1A Fig. (ZIP)
