# Supplementary figures and images for: Hypoxia induced hERG trafficking defect linked to cell cycle arrest in SH-SY5Y cells
Source: PLoS One. 2019 Apr 24;14(4):e0215905. doi: 10.1371/journal.pone.0215905 (PMC6481834; doi:10.1371/journal.pone.0215905)

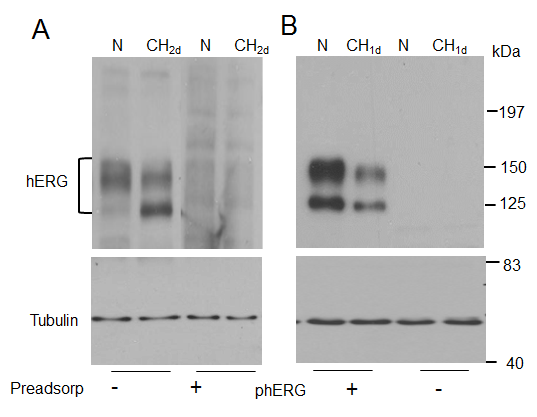

Supplement: S1 Fig — Representative immunoblots showing the specificity of the two hERG protein bands (150 and kDa). A) Diluted hERG antibody preadsorbed with excess of the immunogen (provided with the antibody) overnight was used for immune blot of SH-SY5Y cells exposed to normoxia (N) or 2days of hypoxia (CH2d) and compared to unadsorbed antibody (left two lanes). B) HERG protein expression in HEK cells stably transfected with hERG plasmid subjected to normoxia (N) or 1day of hypoxia (CH1d) and compared with non-transfected HEK cells. Tubulin protein expression was used as a loading control in A and B. (TIF) [file pone.0215905.s001.tif]

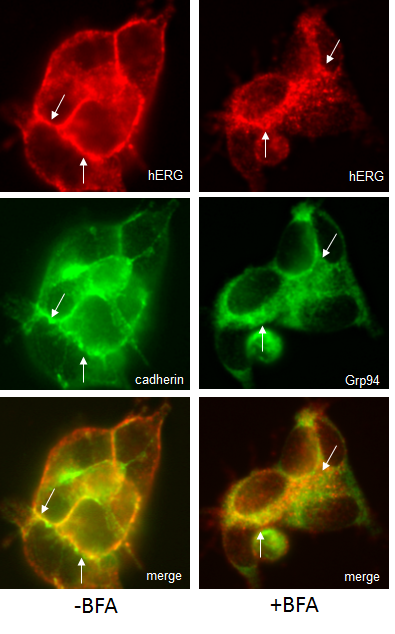

Supplement: S2 Fig — Immunolocalization of hERG (red) with cadherin (green) and Grp94 (green) in SH-SY5Y cells under normoxic conditions with and without brefeldin treatment. Arrows denote membrane localization and ER accumulation of hERG without and with brefeldin treatment respectively. (TIF) [file pone.0215905.s002.tif]
