# Supplementary figures and images for: Correction: Hypoxia induced hERG trafficking defect linked to cell cycle arrest in SH-SY5Y cells
Source: PLoS One. 2024 Jan 11;19(1):e0297301. doi: 10.1371/journal.pone.0297301 (PMC10783753; doi:10.1371/journal.pone.0297301)

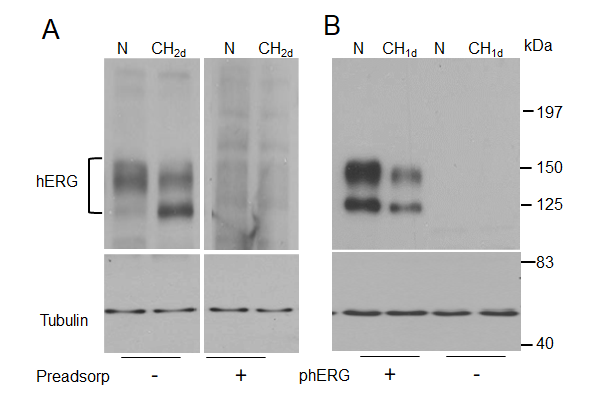

Supplement: S1 Fig — A) Representative immunoblots showing the specificity of the two hERG protein bands (150 and kDa) in SH-SY5Y cells exposed to normoxia (N) or 2days of hypoxia (CH2d) probed with hERG antibody (left two lanes) or with hERG antibody preadsorbed overnight with excess of the immunogen (provided with the antibody) (right two lanes). B) HERG protein expression in HEK cells stably transfected with hERG plasmid subjected to normoxia (N) or 1day of hypoxia (CH1d) and compared with non-transfected HEK cells. Tubulin protein expression was used as a loading control in A and B. (TIF) [file pone.0297301.s001.tif]

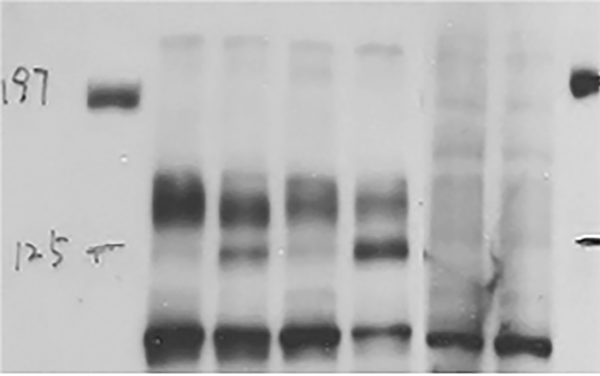

Supplement: S2 File — (ZIP) [file pone.0297301.s003.zip › Fig S1A hERG raw image.tif]

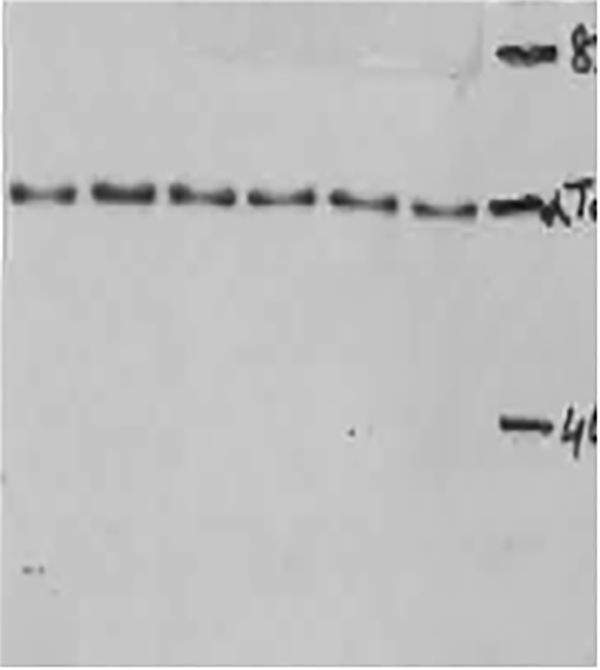

Supplement: S2 File — (ZIP) [file pone.0297301.s003.zip › Fig S1A tubulin raw image.tif]
